# First-Trimester Systemic Immune-Inflammation Index, APRI and FIB-4 Scores in Women Who Later Develop Intrahepatic Cholestasis of Pregnancy: Association with the Disease and with Perinatal Outcomes in a Retrospective Case–Control Study

**DOI:** 10.3390/diagnostics16213610

**Published:** 2026-11-01

**Authors:** Mukremin Ceylan, Mucahit Furkan Balcı, Abdulmecit Öktem, Raziye Torun, Orhan Nural, Hakan Golbasi

**Affiliations:** 1 Department of Perinatology, Health Sciences University, City Hospital of Izmir, Izmir 35540, Turkey; abdulmecitoktem72@gmail.com (A.Ö.); drorhannural@gmail.com (O.N.); drhkngolbasi@gmail.com (H.G.); 2 Department of Obstetrics and Gynecology, Torbalı State Hospital, Izmir 35860, Turkey; mucahitfurkanbalci@gmail.com; 3 Department of Perinatology, Democracy University Buca Seyfi Demirsoy Education and Research Hospital, Izmir 35390, Turkey; drraziyetorun@gmail.com

**Keywords:** intrahepatic cholestasis of pregnancy, systemic immune-inflammation index, APRI, FIB-4, bile acids, first trimester, spectrum bias

## Abstract

**Objective:** To examine whether the systemic immune-inflammation index (SII), neutrophil-to-lymphocyte ratio (NLR), platelet-to-lymphocyte ratio (PLR), AST-to-platelet ratio index (APRI) and fibrosis-4 (FIB-4) score, derived from routine first-trimester tests, are associated with subsequent intrahepatic cholestasis of pregnancy (ICP) and, within ICP, with adverse perinatal outcomes. **Materials and Methods:** This retrospective case–control study included 115 women with ICP and 64 controls with normal liver tests. Indices were derived from 11- to 14-week values. The two primary hypotheses concerned APRI and SII (Bonferroni α = 0.025); all other analyses were exploratory, with false discovery rate (FDR) adjustment. Within ICP, the co-primary outcomes were a composite of preterm birth, meconium-stained amniotic fluid, fetal distress, neonatal intensive care admission or 1-min Apgar score < 7, and the same composite excluding preterm birth, because delivery timing was protocol-driven by bile acids. **Results:** APRI (AUC 0.786; 95% CI 0.718–0.852; *p* < 0.001) and SII (AUC 0.615; 0.530–0.697; *p* = 0.011) were higher in women who later developed ICP; FIB-4 was not. ALT alone discriminated better (AUC 0.895; DeLong *p* = 0.003), APRI and SII added no discrimination beyond ALT (AUC 0.904; *p* = 0.465), and when cases were restricted to the transaminase range of controls (ALT ≤ 31 U/L), the AUC of APRI fell to 0.644. Within ICP, no index demonstrated an association with either composite outcome (all FDR-adjusted q > 0.15), although power was only ~45% for an AUC of 0.60. Bile acids were associated with the composite excluding preterm birth (AUC 0.681; adjusted OR 1.43 per 10 µmol/L; 1.16–1.76); the stronger association with the full composite (AUC 0.754) is partly definitional. **Conclusions:** First-trimester APRI and SII carry a group-level signal in women who later develop ICP, but this signal largely reflects transaminases and is inflated by control selection. The indices did not demonstrate an association with perinatal outcomes, although small effects cannot be excluded; surveillance and delivery timing should continue to rely on bile acids.

## 1. Introduction

Intrahepatic cholestasis of pregnancy (ICP) is the most common pregnancy-specific liver disorder and typically presents in the late second or third trimester with pruritus without rash, elevated serum bile acids, and variable elevation of transaminases. Its prevalence varies markedly between populations, ranging from 0.5% to 1.5% in most European populations and reaching higher rates in South American and Scandinavian populations [1]. The pathogenesis is multifactorial: against a background of genetic susceptibility conferred by variants in genes encoding bile salt transporters (ABCB4, ABCB11, ATP8B1, NR1H4), the rising concentrations of estrogen and progesterone metabolites in advancing gestation impair hepatocellular bile transport; this is accompanied by endoplasmic reticulum stress, mitochondrial dysfunction, alterations in extracellular matrix regulation and activation of immune–inflammatory pathways [2]. ICP is therefore regarded not as a purely cholestatic condition but as a complex process in which hepatocellular injury and a systemic inflammatory response coexist.

Although the maternal course of ICP is largely benign, the fetal risks are considerable. Spontaneous preterm birth, meconium-stained amniotic fluid, fetal distress, neonatal intensive care unit (NICU) admission and, at the most severe end, stillbirth are recognized consequences of the disease. Large meta-analyses based on individual patient data show that these risks are not evenly distributed and that stillbirth risk increases significantly only when total serum bile acids exceed 100 µmol/L [3]. Current guidelines therefore recommend basing the diagnosis on serum bile acid measurement and tailoring the intensity of surveillance and the timing of delivery to bile acid concentrations [4]. This approach, however, carries an inherent delay: bile acids are measured only after pruritus has developed, results may take several days in many centres, and risk stratification can therefore be undertaken only once the disease is already established. An inexpensive, widely available marker applicable in early pregnancy could fill this gap and would have clear clinical value.

Composite indices derived from the complete blood count have been widely studied in obstetric research in recent years because they reflect the systemic inflammatory balance more comprehensively than individual cell counts. Foremost among these is the systemic immune-inflammation index (SII), which integrates neutrophil, platelet, and lymphocyte counts [5]. Similarly, the aspartate aminotransferase-to-platelet ratio index (APRI) and the fibrosis-4 (FIB-4) score, developed for the non-invasive assessment of liver injury and fibrosis, are simple tools calculable from routine laboratory data that have gained acceptance in hepatology as alternatives to biopsy [6]. In ICP, these indices have mostly been examined at diagnosis, in the third trimester, where APRI discriminates ICP from healthy pregnancy and is associated with disease severity [7,8,9,10]. A smaller number of retrospective case–control studies have examined first-trimester APRI as an early marker of later ICP, reporting optimal cut-offs ranging from 0.06 to 0.57 with heterogeneous accuracy [11,12,13,14,15], whereas another study found a significant difference only in the third trimester [16]; early-pregnancy SII has likewise been linked to later ICP in a single cohort [17]. Three questions therefore remain open: whether the first-trimester signal adds anything beyond the transaminases from which APRI is partly derived; how much of the reported accuracy reflects the selection of controls with normal liver tests; and whether first-trimester indices carry any information on perinatal risk once ICP has developed. In this study, we examined the association of first-trimester SII, neutrophil-to-lymphocyte ratio (NLR), platelet-to-lymphocyte ratio (PLR), APRI and FIB-4 with subsequent ICP, including their contribution beyond ALT and their performance in a transaminase-restricted spectrum, and their association with adverse perinatal outcomes among women with ICP.

## 2. Materials and Methods

### 2.1. Study Design and Ethical Approval

This single-center, retrospective case–control study was approved by the Non-Interventional Clinical Research Ethics Committee of İzmir City Hospital (date: 22 July 2026, decision no. 2026/442), and all procedures were performed in accordance with the principles of the Declaration of Helsinki. Because of the retrospective design and the use of anonymized hospital records, the ethics committee waived the requirement for individual informed consent. The study is reported in accordance with the 22-item Strengthening the Reporting of Observational Studies in Epidemiology (STROBE) checklist for case–control studies [18]; because part of the analysis concerns discrimination, risk of bias was additionally appraised with the QUADAS-2 domains [19] (Supplementary Table S3).

### 2.2. Setting and Participants

**Figure 1:**
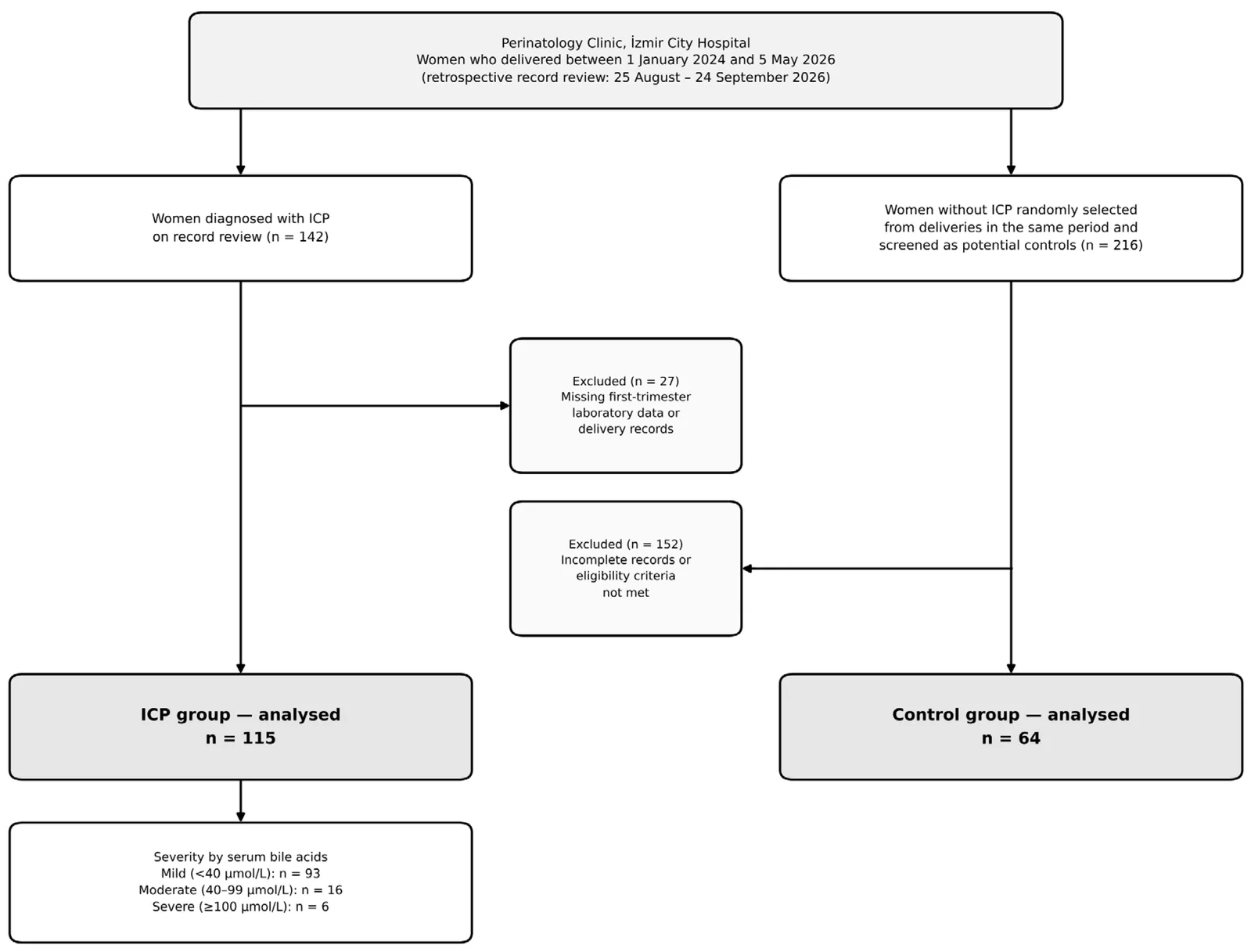
Flow diagram of participant selection. ICP: intrahepatic cholestasis of pregnancy.

The study population comprised women who received antenatal care at the Perinatology Clinic of İzmir City Hospital and delivered at the same center. The hospital information management system and archived records were reviewed retrospectively between 25 August and 24 September 2026, covering women who delivered between 1 January 2024 and 5 May 2026. ICP was diagnosed in the presence of otherwise unexplained pruritus and a fasting total serum bile acid concentration of ≥10 µmol/L; elevated transaminases were recorded as supportive findings [4,20]. We enrolled all women with ICP who met the diagnostic criteria consecutively. The control group was selected by simple random sampling from women who delivered during the same period, had no pruritus, had liver function tests within reference limits in the first trimester and throughout pregnancy, and had no diagnosis of liver disease; cases and controls were not matched. All controls had first-trimester AST and ALT values of ≤31 U/L. The records of 216 women without ICP, randomly selected from those who delivered during the same period, were screened; all 64 women with complete records who met every eligibility criterion were included, and the remaining 152 were excluded because of incomplete records or failure to meet the eligibility criteria. The number of controls was therefore determined by eligibility rather than fixed in advance. Serum bile acids were not measured in asymptomatic controls, in line with routine practice (Figure 1).

Exclusion criteria for both groups were multiple pregnancy; known chronic liver disease (viral hepatitis, autoimmune hepatitis, primary biliary cholangitis, cirrhosis or gallstone disease); pre-eclampsia, HELLP syndrome or acute fatty liver of pregnancy; pre-gestational diabetes, chronic hypertension, chronic kidney disease, hematological disease or malignancy; active infection or inflammatory disease; use of corticosteroids, immunosuppressive or anti-inflammatory drugs during pregnancy; unavailable first-trimester laboratory data; and incomplete delivery records. Figure 1 summarises the participant selection process.

### 2.3. Variables and Definitions

Recorded demographic and obstetric variables were age, gravidity, parity, and body mass index (BMI). BMI was calculated as weight measured at the first antenatal visit divided by height squared. Laboratory variables comprised the complete blood count (neutrophil, lymphocyte and platelet counts) and liver function tests (AST and ALT) obtained from blood samples drawn at the routine first-trimester screening visit at 11–14 weeks of gestation. Total serum bile acids were measured in a fasting sample obtained at diagnosis, before initiation of ursodeoxycholic acid, and were used to grade disease severity in the ICP group.

Adverse perinatal outcomes were defined a priori. Preterm birth was defined as delivery before 37 weeks of gestation; meconium-stained amniotic fluid as the presence of meconium in the amniotic fluid at delivery; fetal distress as a category II/III intrapartum fetal heart rate pattern requiring urgent intervention; and NICU admission as admission of the neonate to the intensive care unit within the first 24 h after birth. The composite adverse perinatal outcome was defined as the presence of at least one of preterm birth, meconium-stained amniotic fluid, fetal distress, NICU admission, or a 1-min Apgar score < 7. Disease severity was classified according to serum bile acid concentration as mild (<40 µmol/L), moderate (40–99 µmol/L) or severe (≥100 µmol/L) [3,20]. According to institutional protocol, delivery is planned at 37 weeks of gestation in women with ICP, and earlier delivery is decided on an individualised basis in women with serum bile acids ≥40 µmol/L; a substantial proportion of preterm births in this series were therefore iatrogenic, which must be considered when interpreting the results. Because the mode of labour onset (spontaneous labour, induction or planned caesarean section) was not systematically recorded, preterm births could not be classified as spontaneous or iatrogenic. As the use of the ≥40 µmol/L bile acid threshold for both severity grading and timing of delivery renders the association between bile acids and preterm birth partly definitional, a second composite outcome excluding the preterm birth component (meconium-stained amniotic fluid, fetal distress, NICU admission or 1-min Apgar score < 7) was defined and analysed as a co-primary outcome within the ICP group.

### 2.4. Calculation of Indices

All indices were calculated from first-trimester laboratory values using the following formulae:

NLR = neutrophil count/lymphocyte count;

PLR = platelet count/lymphocyte count;

SII = (neutrophil count × platelet count)/lymphocyte count [5];

APRI = [AST (U/L)/upper limit of normal for AST (40 U/L)] × 100/platelet count (10^9^/L) [6].

FIB-4 = [age (years) × AST (U/L)]/[platelet count (10^9^/L) × √ALT (U/L)] [21].

The AST/ALT (De Ritis) ratio, which reflects the pattern of hepatocellular injury, was also calculated. All blood counts were measured on an automated haematology analyser (XN-1000; Sysmex Corporation, Kobe, Japan), and biochemical parameters by standard autoanalyser methods (Cobas c702; Roche Diagnostics, Mannheim, Germany) in the same central laboratory.

### 2.5. Outcome Measures and Hypotheses

The primary outcome was the association of first-trimester APRI and SII, the two indices named in the study protocol approved by the ethics committee, with subsequent ICP, quantified as the area under the receiver operating characteristic curve (AUC). These two primary hypotheses were tested at a Bonferroni-corrected α of 0.025 each. Because the case–control design with healthy controls does not reproduce the population in which a screening test would be used, these AUCs are interpreted as measures of group-level association rather than of clinical diagnostic accuracy. Within the ICP group, the composite adverse perinatal outcome and the composite excluding preterm birth were co-primary outcomes. All other analyses (other indices, preterm birth and NICU admission as single outcomes, severity subgroups, correlations and sensitivity analyses) were secondary and are regarded as exploratory.

### 2.6. Control of Bias

To minimize selection bias, we enrolled all women with ICP who met the diagnostic criteria. Data were extracted independently by two investigators using a standardized data collection form, and discrepancies were resolved by reference to the source records. Laboratory values were recorded according to a predefined time window (11–14 weeks) and separately from outcome data. Because BMI differed significantly between groups, all perinatal outcome analyses were repeated using logistic regression models adjusted for BMI and age to control for potential confounding. Because the selection of controls with normal liver tests may inflate the discrimination of transaminase-based markers (spectrum bias) [22,23], the analysis of ICP versus controls was repeated after restricting cases to women whose first-trimester ALT lay within the range observed in controls (≤31 U/L).

### 2.7. Sample Size

Because all eligible women during the study period were included, no a priori sample size calculation was performed. Instead, the sensitivity of the available sample was assessed in terms of the smallest detectable effect using the Hanley–McNeil approach [24]: with α = 0.05 (two-sided), the sample of 115 cases and 64 controls had approximately 93% power to distinguish an area under the curve (AUC) of 0.65 from 0.50. For the predictive analyses within the ICP group (52 events vs. 63 non-events), power was approximately 80% for an AUC of 0.65 and approximately 45% for an AUC of 0.60; these analyses are therefore insufficient to exclude small effects. The unequal number of cases and controls (approximately 1.8:1) reduces statistical efficiency relative to a balanced design but does not bias the AUC, sensitivity, specificity or odds ratios, which are conditional on group membership [24,25]; only predictive values depend on the case proportion and were therefore recalculated for a population prevalence. Post hoc power analysis was not performed, as it merely re-expresses the observed *p*-values.

### 2.8. Statistical Analysis

Data were analysed using SPSS version 27.0 (IBM Corp., Armonk, NY, USA); bootstrap confidence intervals for ROC curves, DeLong tests, variance inflation factor (VIF) calculations, false discovery rate adjustment, Hosmer–Lemeshow tests, resampling analyses, and figures were produced with Python version 3.13 (Python Software Foundation, Wilmington, DE, USA) using the SciPy (version 1.18), statsmodels (version 0.15), scikit-learn (version 1.9), and matplotlib (version 3.11) libraries. Normality of continuous variables was assessed with the Shapiro–Wilk test; because no continuous variable was normally distributed in both groups, continuous data are presented as median (interquartile range, IQR) and compared with the Mann–Whitney U test; the Kruskal–Wallis test was used for three-group comparisons, with Bonferroni-corrected pairwise comparisons where the overall test was significant. In the severe subgroup (*n* = 6), medians are accompanied by the range, and individual values are given in Supplementary Table S1. Categorical variables are presented as *n* (%), with Wilson 95% confidence intervals for subgroup proportions, and were compared using the chi-square, Fisher’s exact, or Fisher–Freeman–Halton tests. Group differences in perinatal outcomes are expressed as odds ratios (OR) with 95% confidence intervals (CI) and Wald *p*-values obtained from the same logistic regression model, so that each OR, CI, and *p*-value derives from a single framework. The between-group difference in birth weight was re-examined using linear regression adjusted for gestational age at delivery (ANCOVA).

Discrimination was evaluated using receiver operating characteristic (ROC) curves; 95% CIs for AUCs were computed with 4000 bootstrap replicates, and AUCs derived from the same subjects were compared using the DeLong test. The *p*-value for each AUC (null hypothesis AUC = 0.5) was obtained from the Mann–Whitney U test, which is mathematically equivalent, so that *p*-values in the descriptive and ROC tables coincide. All AUCs are reported in the a priori hypothesised direction (higher values indicating a higher probability of ICP or of the adverse outcome; for the AST/ALT ratio, lower values, reflecting ALT-predominant injury); no curve was re-oriented post hoc, so an AUC below 0.5 indicates an inverse association. Optimal cut-off values were determined by the Youden index, at which sensitivity, specificity, positive predictive value (PPV), negative predictive value (NPV) and accuracy were calculated; cut-offs are reported only for markers whose AUC differed significantly from 0.5 in the hypothesised direction. Because PPV and NPV depend on the proportion of ICP in the sample (64.2%), these values were additionally recalculated for a population prevalence of 1% using Bayes’ theorem. The incremental contribution of the derived indices beyond transaminases was tested by adding APRI and SII to a model containing ALT alone, using the likelihood ratio test and AUC comparison. Correlations between continuous variables were assessed with Spearman’s rank correlation coefficient (ρ).

Covariates in the multivariable logistic regression models were specified a priori: age, BMI, APRI and SII for the model of ICP presence, and bile acids, APRI, SII, age and BMI for the perinatal outcome models within the ICP group. In every model, the dependent variable was binary, and the reference category is stated explicitly: ICP (coded 1) versus control (coded 0, reference), or, within the ICP group, the presence (1) versus absence (0, reference) of the respective outcome. All predictors were entered as continuous variables, so there is no reference group for the predictors; ORs express the change in odds per 1-standard-deviation (SD) increase, which places indices measured on different scales on a comparable footing. Bile acids are expressed per 10 µmol/L increase instead, because this unit corresponds to guideline thresholds and because the SD of this right-skewed variable (26.8 µmol/L) is dominated by a few very high values. Because NLR is a mathematical component of SII, it was not entered into the main models; models including NLR and alternative models including all variables with *p* < 0.20 in univariable analysis were fitted as sensitivity analyses. Multicollinearity was assessed with the VIF (VIF > 5 considered problematic), model fit with Nagelkerke R^2^, and calibration with the Hosmer–Lemeshow test (10 groups) [26].

Multiplicity was handled as follows. The two primary hypotheses were tested at α = 0.025. For all secondary inferential tests, Benjamini–Hochberg false discovery rate (FDR)-adjusted q values are reported [27], computed within two families: the baseline comparisons in Table 1 (15 tests), and the ROC and regression analyses of the secondary markers and outcomes (39 tests; listed in Supplementary Table S4). Findings with q ≥ 0.05 are described as not supported after correction. Prespecified sensitivity analyses were: (i) redefinition of ICP using the ≥19 µmol/L threshold of the Royal College of Obstetricians and Gynaecologists (RCOG) [28]; (ii) the transaminase-restricted analysis described above; (iii) addition of ALT to the multivariable model of ICP presence; and (iv) 2000 random 1:1 subsamples of 64 cases and 64 controls, to examine the influence of the unequal group sizes. A *p*-value < 0.05 was considered statistically significant unless otherwise stated.

## 3. Results

### 3.1. Characteristics of the Study Population

Of the 142 women identified with ICP on record review, 27 (19.0%) with missing first-trimester laboratory data or delivery records were excluded; of the 216 women screened for the control group, 64 met all eligibility criteria (Figure 1). Consequently, 115 women with ICP and 64 healthy pregnant women without liver disease selected by simple random sampling were included in the analysis, giving a total study population of 179. The groups were similar in age (median 30.0 [IQR 26.0–33.0] vs. 29.0 [25.0–34.0] years; *p* = 0.469), gravidity (*p* = 0.827) and parity (*p* = 0.172). In contrast, BMI was significantly lower in the ICP group (28.4 [27.0–30.5] vs. 30.1 [28.6–32.0] kg/m^2^; *p* = 0.004; q = 0.009); BMI was therefore included as a potential confounder in subsequent models (Table 1, Panel A). No stillbirths occurred in either group, and all neonates had a 1-min Apgar score ≥5.

### 3.2. Laboratory Parameters and Systemic Inflammatory/Fibrosis Indices

As expected, transaminases were markedly higher in the ICP group: AST 25.0 (19.5–33.0) vs. 17.0 (13.0–21.2) U/L and ALT 33.0 (20.5–45.5) vs. 13.0 (12.0–15.0) U/L (both *p* < 0.001). Accordingly, the AST/ALT (De Ritis) ratio was significantly lower in the ICP group (0.83 [0.62–0.97] vs. 1.27 [0.93–1.64]; *p* < 0.001), which was interpreted as indicating predominantly ALT-driven hepatocellular injury in ICP. First-trimester ALT exceeded 40 U/L in 41 women with ICP (35.7%), whereas no control exceeded 31 U/L.

**Figure 2:**
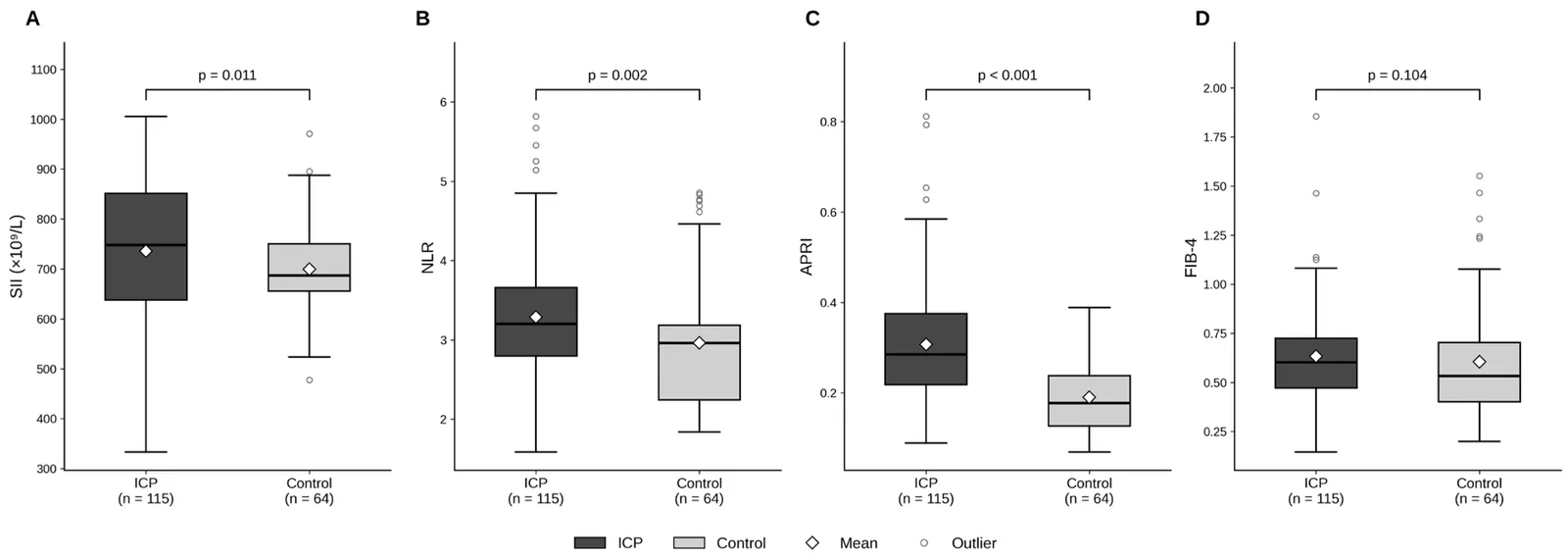
Distribution of first-trimester systemic immune-inflammation index (SII (**A**)), neutrophil-to-lymphocyte ratio (NLR (**B**)), APRI (**C**), and FIB-4 (**D**) in the ICP and control groups. Boxes indicate the interquartile range (Q1–Q3), the thick central line the median, and the diamond the mean; whiskers extend to the most extreme observations within 1.5× IQR, and observations beyond this limit are shown as separate points. *p*-values are from the Mann–Whitney U test. APRI: AST-to-platelet ratio index; FIB-4: fibrosis-4 index.

Among systemic inflammatory markers, neutrophil count (6.37 [5.63–7.48] vs. 6.00 [5.80–6.20] × 10^9^/L; *p* = 0.026; q = 0.049), NLR (3.20 [2.80–3.66] vs. 2.96 [2.24–3.18]; *p* = 0.002; q = 0.005) and SII (747.8 [638.0–851.2] vs. 686.7 [655.5–750.2]; *p* = 0.011) were significantly higher in the ICP group. Lymphocyte count (*p* = 0.130), platelet count (*p* = 0.170), and PLR (*p* = 0.455) did not differ between groups. Among the liver fibrosis indices, APRI was markedly higher in the ICP group (0.285 [0.218–0.376] vs. 0.178 [0.127–0.238]; *p* < 0.001), whereas FIB-4 did not differ significantly (0.60 [0.47–0.72] vs. 0.53 [0.40–0.70]; *p* = 0.104) (Table 1, Panel C; Figure 2). Both primary hypotheses were therefore supported at the Bonferroni-corrected α of 0.025.

### 3.3. Perinatal Outcomes

Gestational age at delivery was significantly lower in the ICP group (37.0 [36.3–37.0] vs. 38.0 [37.0–39.0] weeks; *p* < 0.001), as was birth weight (2950 [2750–3120] vs. 3175 [2973–3413] g; *p* < 0.001). The 1-min Apgar score was also lower in the ICP group (*p* = 0.002), although the clinical relevance of this difference was limited, as the proportion with an Apgar score < 7 was similar in the two groups (4.3% vs. 4.7%; OR 0.92; *p* = 0.916). However, the difference in birth weight disappeared after adjustment for gestational age at delivery (adjusted difference −26 g; 95% CI −130 to 78; *p* = 0.620) and remained unchanged after further adjustment for age and BMI (−37 g; *p* = 0.495), indicating that the lower birth weight in the ICP group largely reflected earlier delivery.

With regard to categorical perinatal outcomes, the rate of preterm birth was approximately twice as high in the ICP group (33.9% vs. 15.6%; OR 2.77; 95% CI 1.27–6.03; *p* = 0.010). Meconium-stained amniotic fluid was numerically more frequent in the ICP group (13.9% vs. 6.3%), but the difference was not statistically significant (OR 2.42; 0.77–7.59; *p* = 0.128). Rates of fetal distress (13.9% vs. 10.9%; *p* = 0.569) and NICU admission (23.5% vs. 23.4%; *p* = 0.995) were similar between groups. The composite outcome occurred in 45.2% of the ICP group and 32.8% of controls (OR 1.69; 95% CI 0.89–3.20; *p* = 0.107), and the composite excluding preterm birth in 27.8% and 25.0%, respectively (OR 1.16; 0.58–2.32; *p* = 0.683) (Table 2).

In view of the BMI difference between groups, logistic regression models adjusted for BMI and age were fitted for all perinatal outcomes. Effect sizes were virtually unchanged after adjustment: the adjusted OR was 2.71 (95% CI 1.23–6.00; *p* = 0.014) for preterm birth and 1.56 (95% CI 0.81–3.01; *p* = 0.182) for the composite adverse outcome. Results were likewise unchanged for meconium-stained amniotic fluid (adjusted OR 2.26; *p* = 0.171), fetal distress (adjusted OR 1.15; *p* = 0.781), NICU admission (adjusted OR 0.93; *p* = 0.856) and the composite excluding preterm birth (adjusted OR 1.09; *p* = 0.812). BMI was not an independent predictor in any model (all *p* > 0.15) (Table 2).

### 3.4. Discrimination of ICP from Controls by First-Trimester Markers

In ROC analysis, ALT showed, as expected, the best discrimination between ICP and controls (AUC 0.895; 95% CI 0.843–0.940; *p* < 0.001; cut-off ≥20 U/L, sensitivity 75.7%, specificity 96.9%). Among the derived markers, APRI (AUC 0.786; 95% CI 0.718–0.852; *p* < 0.001) and the AST/ALT ratio (AUC 0.787; 95% CI 0.712–0.853; *p* < 0.001) performed best, with nearly identical values. At a cut-off of ≥0.245, APRI had a sensitivity of 67.0%, specificity of 79.7%, PPV of 85.6%, and an accuracy of 71.5% (positive likelihood ratio 3.3; negative likelihood ratio 0.41); the AST/ALT ratio provided the same discrimination in the opposite direction (≤0.977: sensitivity 75.7%, specificity 73.4%). By the DeLong test, the AUC of ALT was significantly higher than that of APRI (*p* = 0.003); APRI did not differ from the AST/ALT ratio (*p* = 0.992) or AST (*p* = 0.869), but discriminated significantly better than SII (*p* = 0.004) and NLR (*p* = 0.001). The PPV of 85.6%, which depends on the proportion of ICP in the sample (64.2%), falls to 3.2% at an assumed population prevalence of 1%, with an NPV of 99.6%.

**Figure 3:**
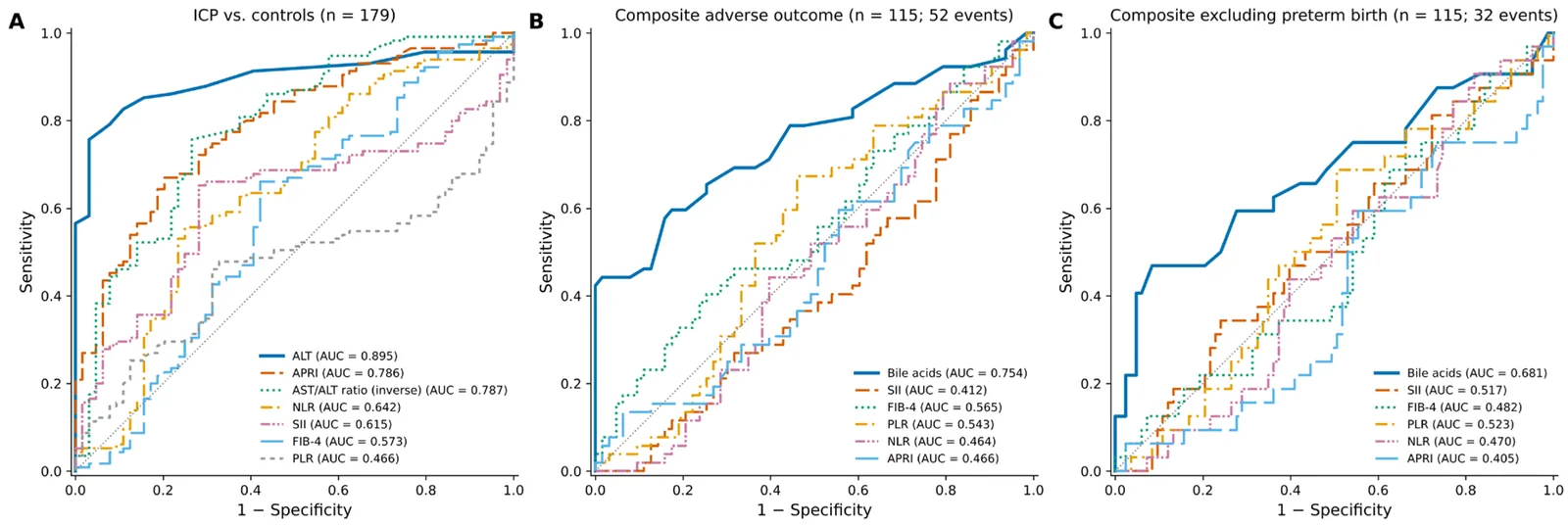
Receiver operating characteristic (ROC) curves. (**A**) Discrimination of ICP from controls (*n* = 179); (**B**) composite adverse perinatal outcome in women with ICP (*n* = 115); (**C**) composite outcome excluding preterm birth in women with ICP (*n* = 115). All curves are drawn in the a priori hypothesised direction (higher values indicating a higher probability of the event; inverse for the AST/ALT ratio), so curves below the diagonal (AUC < 0.5) indicate inverse associations. The dashed diagonal line indicates no discrimination. AUC: area under the curve.

The discrimination of the inflammatory indices was statistically significant but clinically weak: AUC 0.642 (95% CI 0.552–0.726; *p* = 0.002; q = 0.011) for NLR and 0.615 (95% CI 0.530–0.697; *p* = 0.011) for SII. The AUCs for FIB-4 (0.573; *p* = 0.104) and PLR (0.466; *p* = 0.455) did not differ significantly from 0.5 (Table 3, Panel A; Figure 3A). When the incremental contribution of the derived indices beyond transaminases was tested, adding APRI and SII to a model containing ALT alone modestly improved model fit (likelihood ratio test *p* = 0.032) but did not significantly improve discrimination (AUC from 0.895 to 0.904; DeLong *p* = 0.465).

Sensitivity analyses. When cases were restricted to the 50 women whose first-trimester ALT was ≤31 U/L, the range observed in controls, the AUC fell from 0.895 to 0.759 for ALT and from 0.786 to 0.644 (95% CI 0.541–0.741) for APRI, whereas the AUCs of SII (0.621; 0.508–0.738) and NLR (0.641; 0.534–0.742), which contain no transaminase, were essentially unchanged. When ICP was redefined with the RCOG threshold of ≥19 µmol/L (89 cases; 26 women reclassified), APRI (AUC 0.782; 0.708–0.849), SII (0.597; 0.508–0.689; *p* = 0.041) and ALT (0.897) behaved as in the main analysis. In 2000 random 1:1 subsamples of 64 cases and 64 controls, the median AUCs were virtually identical to the full-sample estimates (APRI 0.787 [2.5th–97.5th percentile 0.746–0.826]; SII 0.614 [0.557–0.675]; NLR 0.642; ALT 0.896); APRI was significant in 100% of subsamples and SII in 67%, reflecting the loss of power with fewer cases rather than any change in effect size (Supplementary Table S2).

### 3.5. Adverse Perinatal Outcomes Within the ICP Group

Within the ICP group, women with (*n* = 52; 45.2%) and without (*n* = 63; 54.8%) the composite adverse perinatal outcome did not differ significantly in any inflammatory or fibrosis index: NLR (3.21 [2.81–3.55] vs. 3.20 [2.80–3.75]; *p* = 0.504), SII (739.8 [620.6–824.9] vs. 761.4 [684.4–865.8]; *p* = 0.105), APRI (0.278 [0.224–0.364] vs. 0.301 [0.218–0.386]; *p* = 0.527) and FIB-4 (0.60 [0.50–0.78] vs. 0.60 [0.46–0.68]; *p* = 0.230). In contrast, serum bile acids were markedly higher in women with an adverse outcome (34.0 [23.0–49.5] vs. 21.0 [17.0–28.0] µmol/L; *p* < 0.001). These women also delivered earlier (36.0 [36.0–36.9] vs. 37.0 [37.0–37.0] weeks; *p* < 0.001) and had lower birth weights (2805 [2600–3000] vs. 3000 [2900–3200] g; *p* < 0.001) (Table 4).

### 3.6. Disease Severity Subgroups According to Serum Bile Acids

Based on serum bile acids, women were classified as having mild (<40 µmol/L; *n* = 93; 80.9%), moderate (40–99 µmol/L; *n* = 16; 13.9%), or severe (≥100 µmol/L; *n* = 6; 5.2%) disease. NLR (*p* = 0.816), SII (*p* = 0.349), APRI (*p* = 0.083) and FIB-4 (*p* = 0.054) did not differ significantly across severity groups; only ALT increased with severity (median 33.0, 39.0 and 71.0 U/L; *p* = 0.024; Bonferroni-adjusted *p* = 0.043 for mild vs. severe).

**Figure 4:**
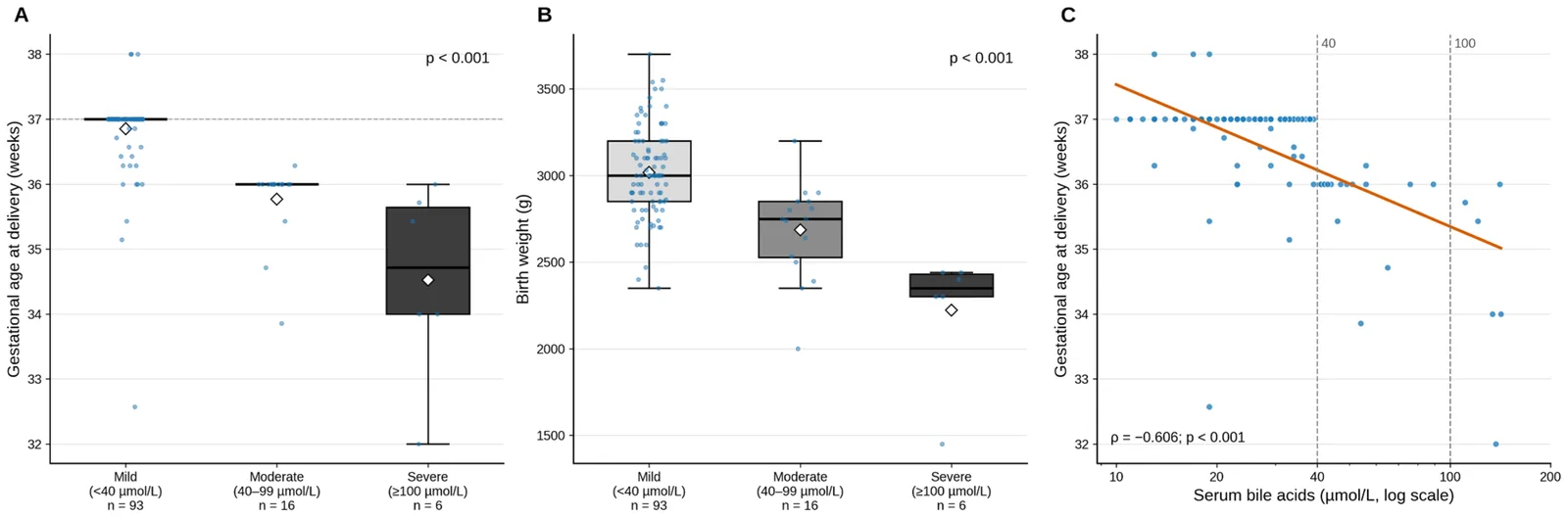
Gestational age at delivery (**A**) and birth weight (**B**) across disease severity groups defined by serum bile acids. Boxes indicate the interquartile range, the thick line the median, the diamond the mean, and dots individual women; whiskers extend to the most extreme observations within 1.5× IQR. *p*-values are from the Kruskal–Wallis test; the dashed horizontal line in (**A**) marks 37 weeks of gestation. (**C**) Relationship between serum bile acids and gestational age at delivery; the x-axis is logarithmic, the orange line shows the log-linear fit, and the dashed vertical lines mark the severity thresholds of 40 and 100 µmol/L. The clustering of deliveries at 37 and 36 weeks reflects the institutional delivery-timing protocol. ρ: Spearman correlation coefficient.

In contrast, perinatal outcomes showed a strong, graded association with severity. Gestational age at delivery declined significantly from mild to severe disease (median 37.0, 36.0 and 34.7 weeks; *p* < 0.001), with all pairwise comparisons significant. Birth weight followed a similar pattern (3000, 2750 and 2350 g; *p* < 0.001), and the difference persisted after adjustment for gestational age at delivery (−194 g for moderate, *p* = 0.018, and −499 g for severe disease, *p* < 0.001, relative to mild disease). The 1-min Apgar score also decreased with increasing severity (*p* = 0.002), and the proportion with an Apgar score < 7 rose from 0% in the mild group to 18.8% and 33.3% in the moderate and severe groups, respectively (*p* < 0.001). The preterm birth rate was 18.3% in the mild group and 100% in both the moderate and severe groups (*p* < 0.001). Rates of meconium-stained amniotic fluid (7.5%, 31.3% and 66.7%), fetal distress (5.4%, 43.8% and 66.7%), NICU admission (14.0%, 62.5% and 66.7%) and the composite adverse outcome (32.3%, 100% and 100%) all increased significantly with severity (all *p* < 0.001) (Table 5; Figure 4A,B). Because the severe group comprised only six women, the 95% confidence intervals of these proportions are wide (e.g., 30.0–90.3% for 66.7%; Table 5), and these estimates should be regarded as hypothesis-generating; individual values are listed in Supplementary Table S1.

### 3.7. Correlations of Bile Acids with Indices and Perinatal Outcomes

On Spearman correlation analysis within the ICP group, serum bile acids were not significantly correlated with any inflammatory or fibrosis index: NLR (ρ = −0.046; *p* = 0.622), SII (ρ = −0.093; *p* = 0.321), APRI (ρ = 0.071; *p* = 0.451), FIB-4 (ρ = 0.002; *p* = 0.981) and PLR (ρ = 0.069; *p* = 0.461). The correlation with ALT was of borderline significance (ρ = 0.179; *p* = 0.056). In contrast, bile acids were significantly and negatively correlated with gestational age at delivery (ρ = −0.606; *p* < 0.001), birth weight (ρ = −0.467; *p* < 0.001) and 1-min Apgar score (ρ = −0.259; *p* = 0.005) (Figure 4C). Among the indices, the expected moderate correlations were observed between APRI and FIB-4 (ρ = 0.536) and between SII and NLR (ρ = 0.496).

### 3.8. ROC Analysis of Perinatal Outcomes Within the ICP Group

For the co-primary composite outcome excluding preterm birth (*n* = 32; 27.8%), bile acids showed significant but limited discrimination (AUC 0.681; 95% CI 0.556–0.796; *p* = 0.003; q = 0.015; ≥41 µmol/L: sensitivity 46.9%, specificity 91.6%) and remained independent after adjustment for APRI, SII, age and BMI (OR 1.43 per 10 µmol/L; 95% CI 1.16–1.76; *p* = 0.001). None of the inflammatory or fibrosis indices demonstrated an association with this outcome: SII 0.517, NLR 0.470, PLR 0.523, FIB-4 0.482 and APRI 0.405 (all *p* > 0.10; all q > 0.15) (Table 3, Panel C; Figure 3C). The inverse adjusted association of APRI (OR 0.56 per SD; *p* = 0.048) did not survive FDR correction (q = 0.156).

For the full composite outcome, bile acids showed stronger discrimination (AUC 0.754; 95% CI 0.656–0.842; *p* < 0.001). At the Youden-derived cut-off of ≥39 µmol/L, sensitivity was 44.2%, specificity 98.4%, PPV 95.8% and NPV 68.1%; however, because women with bile acids ≥40 µmol/L were delivered before 37 weeks by protocol, this cut-off essentially reproduces the institutional threshold, and the specificity is largely definitional. None of the inflammatory or fibrosis indices demonstrated an association with the full composite outcome: SII (AUC 0.412; *p* = 0.105), FIB-4 (0.565; *p* = 0.230), PLR (0.543; *p* = 0.428), NLR (0.464; *p* = 0.504) and APRI (0.466; *p* = 0.527) (all q > 0.25) (Table 3, Panel B; Figure 3B). Among the 76 women who delivered at or after 37 weeks, all of whom had bile acids <40 µmol/L, bile acids were not associated with the composite excluding preterm birth (AUC 0.49; *p* = 0.912).

In exploratory analyses of single outcomes, bile acids were strongly associated with preterm birth (AUC 0.843; 95% CI 0.756–0.917; *p* < 0.001; ≥39 µmol/L: sensitivity 59.0%, specificity 98.7%), an association that is again largely protocol-driven, and with NICU admission (AUC 0.678; 0.540–0.806; *p* = 0.005; q = 0.025). SII showed an inverse association with preterm birth (AUC 0.354; *p* = 0.010); this finding did not survive Bonferroni correction (p_Bonf_ = 0.41) and was borderline after FDR correction (q = 0.045), and we regard it as a probable chance finding (Table 3, Panel D). Of the 39 preterm births in the ICP group, 36 (92.3%) were late preterm (34 + 0 to 36 + 6 weeks), and only three occurred before 34 weeks; 16 of the 17 preterm births in the mild group occurred after 35 weeks.

### 3.9. Multivariable Logistic Regression Analyses

In the model of ICP presence (dependent variable: ICP = 1 vs. control = 0, reference; *n* = 179), each 1-SD increase in APRI (0.129 units) was associated with 4.79-fold higher odds of ICP (95% CI 2.70–8.50; *p* < 0.001) and each 1-SD increase in SII (131.6 × 10^9^/L) with 1.64-fold higher odds (95% CI 1.11–2.43; *p* = 0.014; q = 0.050). BMI showed an inverse association (OR 0.65 per SD; 0.44–0.95; *p* = 0.027), whereas age was not significant (*p* = 0.089). The model was significant overall (*p* < 0.001; Nagelkerke R^2^ = 0.380; AUC 0.818) and well calibrated (Hosmer–Lemeshow χ^2^ = 7.97; *p* = 0.436), with no evidence of multicollinearity (all VIF ≤ 1.08). In a sensitivity analysis additionally including NLR (all VIF ≤ 1.55), APRI (OR 6.62 per SD) and SII (OR 2.03 per SD) remained independent, whereas NLR, which was positively associated with ICP in univariable analysis, reversed direction (OR 0.64 per SD; *p* = 0.064); this sign reversal was interpreted as a suppression effect arising from the mathematical inclusion of NLR within SII. When ALT was added to the main model, the associations of APRI (OR 2.21 per SD; 1.00–4.87; *p* = 0.049) and SII (OR 1.68 per SD; 1.00–2.83; *p* = 0.050) were attenuated to borderline significance, in keeping with the ROC findings (Table 6, Panel A; Supplementary Table S2).

In the prespecified model for the composite adverse perinatal outcome in the ICP group (dependent variable: outcome present = 1 vs. absent = 0, reference), bile acids remained the only independent predictor: OR 2.38 per 10 µmol/L increase (95% CI 1.54–3.69; *p* < 0.001). SII (OR 0.68 per SD; *p* = 0.092), APRI (OR 0.75 per SD; *p* = 0.239), age (*p* = 0.122) and BMI (*p* = 0.981) did not contribute significantly (Nagelkerke R^2^ = 0.384; Hosmer–Lemeshow *p* = 0.399; all VIF ≤ 1.07) (Table 6, Panel B). The result was unchanged in an alternative model including all variables with *p* < 0.20 in univariable analysis (bile acids, age, FIB-4, SII and NLR) together with BMI: the OR for bile acids was 2.30 (95% CI 1.49–3.56; *p* < 0.001), and no other variable was significant (FIB-4 *p* = 0.107; SII *p* = 0.956; NLR *p* = 0.181). The corresponding model for the composite excluding preterm birth is shown in Table 6, Panel C (Hosmer–Lemeshow *p* = 0.660). In the exploratory model for preterm birth (Table 6, Panel D), bile acids (OR 3.89 per 10 µmol/L; 2.18–6.91) and an inverse association of SII (OR 0.51 per SD; 0.30–0.87; *p* = 0.014; q = 0.050) were observed (Nagelkerke R^2^ = 0.551; Hosmer–Lemeshow *p* = 0.789).

## 4. Discussion

In this study, we showed that routine laboratory data obtained as early as 11–14 weeks of gestation in women who subsequently developed ICP carried a measurable hepatocellular and inflammatory signature months before the disease became clinically apparent. First-trimester APRI was approximately 60% higher in the ICP group than in controls, and SII and NLR also differed significantly, albeit with small effects (AUC 0.62–0.64). In multivariable analysis, APRI and SII remained associated with ICP after adjustment for age and BMI; however, this signal largely reflected transaminase elevation, added no discrimination beyond ALT alone, and was inflated by the selection of controls with normal liver tests: in a transaminase-restricted spectrum, the AUC of APRI fell to 0.644. The second, and clinically decisive, finding of the study is negative: none of these indices demonstrated an association with either co-primary perinatal outcome, with preterm birth or with NICU admission among women with ICP; they were unrelated to disease severity and uncorrelated with serum bile acids. Because the power to detect an AUC of 0.60 was only about 45%, this negative finding excludes moderate but not small associations. As expected, the only variable associated with perinatal risk was serum bile acid concentration.

Our APRI findings should be placed alongside five earlier case–control studies of first-trimester APRI. Tolunay et al. (37 cases, 66 controls) reported a cut-off of 0.57 with 86.5% sensitivity and 77.3% specificity [11]; Gök et al. (49 vs. 62) an AUC of 0.758 at a cut-off of 0.148 (sensitivity 79.6%, specificity 56.5%) [12]; Peker et al. (198 vs. 204) a first-trimester cut-off of 0.101 (sensitivity 79.7%, specificity 79.6%) [13]; and Bozbay et al. (54 vs. 59) an AUC of 0.632 at a cut-off of 0.06, using AST divided by platelet count without normalisation to the upper limit of normal [14]. Obut et al. likewise found higher first-trimester APRI in ICP [15], whereas Saadi et al. reported a significant difference only in the third trimester [16]. Our AUC of 0.786 and cut-off of 0.245 lie within the published range. The almost tenfold spread of published cut-offs (0.06–0.57) reflects differences in the formula (normalised or unnormalised AST), the assumed upper limit of normal, the bile acid threshold used to define ICP and, above all, control selection, and indicates that no first-trimester APRI threshold is yet transportable between centres. The present study adds three elements that these reports did not address: a formal test of incremental value over ALT, which showed none; a transaminase-restricted analysis that quantifies spectrum bias; and an analysis of perinatal outcomes within ICP that accounts for bile acid-driven delivery timing.

Our findings regarding the inflammatory indices are consistent with the scattered and partly conflicting literature. In a retrospective study at Ankara Etlik City Hospital comparing 121 women with ICP and 121 controls, Özkan et al. found SII and NLR to be significantly higher in ICP, but the AUC for SII was only 0.581 [29]; this value is almost identical to the 0.615 we obtained from first-trimester rather than third-trimester measurements. Ergani and Celen, following 218 pregnant women from the first trimester to delivery to examine the course of SII across all three trimesters, reported that the difference became evident in second-trimester SII, which was particularly high in severe ICP, and that a second-trimester SII cut-off identified ICP with 92% sensitivity and 96% specificity [30]; the small effect size we observed in the first trimester is consistent with this observation. Çallıoğlu et al., comparing 173 women with ICP and 266 controls, suggested that elevated SII supports the inflammatory nature of ICP but does not contribute to diagnosis or severity assessment, whereas PLR may be useful [31]. In contrast, in a recent study by Kurt and Yılmaz of 186 cases and 244 controls with indices calculated at diagnosis, the aggregate index of systemic inflammation (AISI), NLR, SII and SIRI did not differ between groups, while neutrophil and lymphocyte counts were lower and PLR higher in ICP [32]. This contradictory result illustrates how markedly the behaviour of haemogram-based indices in ICP varies between studies. Ji et al., comparing 113 women with ICP and 209 controls, reported NLR as an independent factor for ICP (OR 1.885) that was higher in severe than in mild disease [33]; in our series, NLR did not differ across severity groups (*p* = 0.816), a discrepancy that may be explained by our measurements having been obtained before the disease developed.

Earlier studies show a similar heterogeneity. In the series of Yayla Abide et al. comparing 84 women with ICP and 145 healthy pregnant women, NLR did not differ between groups, whereas leucocyte count, mean platelet volume (MPV), and PLR were higher and RDW lower, with MPV being the only marker associated with severity [34]. In their small series of 33 cases and 33 controls, Silva et al. found no difference in any complete blood count parameter in the first trimester, with differences emerging only in the third trimester and, moreover, in the unexpected direction (lower leucocyte and neutrophil counts and NLR) [35]. The study by Shen et al., which compared 49 women with ICP with 250 healthy pregnant women at disease onset and with 1439 in early pregnancy, has the design closest to ours and directly supports our findings: the authors reported that, in early pregnancy before disease onset, platelet parameters such as plateletcrit and PDW, as well as neutrophil count, NLR, SIRI and SII, were significantly higher in women who later developed ICP, and that SII was an independent risk factor for the development of ICP [17]. In the study by Ozgen et al. of 80 cases and 120 controls, NLR and platelet count were higher and RDW lower, but the strongest discrimination was provided by fibrinogen, a coagulation parameter rather than an inflammatory marker [36]. Taken together, these heterogeneous results suggest that haemogram-based indices reflect a real but small inflammatory signal in ICP that lacks the discriminative power required for individual diagnostic decisions. The AUCs of 0.61–0.64 obtained for SII and NLR in our data, which were unchanged in the transaminase-restricted analysis, support precisely this interpretation.

Our findings on the fibrosis indices rest on a more consistent body of literature. Obut et al., comparing 80 women with ICP and 200 controls and measuring markers both in the first trimester and at diagnosis, showed that ALBI, MELD and APRI scores were significantly higher in ICP at both time points, and ALBI, MELD and APRI, together with age, were significant in multivariable logistic regression [15]. This result provides independent confirmation of our first-trimester APRI finding. In a prospective study of 71 women with ICP and 71 controls, Cemortan et al. reported both APRI and FIB-4 as reliable markers associated with major complications of the disease [8]. Eyisoy et al. reported that, in 101 women with ICP, APRI calculated at diagnosis distinguished severe from mild disease with 82% sensitivity and 72% specificity at a cut-off of 1.06, was moderately correlated with fasting bile acids (r = 0.445), and was associated with meconium-stained amniotic fluid, NICU admission, and preterm birth [7]. In the retrospective study by Cicek et al. of 105 women with ICP and 112 controls, APRI calculated at diagnosis discriminated ICP with an AUC of 0.902, and FIB-4 also showed a smaller but significant difference [9]; Yılmaz et al. similarly reported an AUC of 0.842 at a cut-off of 0.295 for third-trimester APRI [10]. The finding that APRI performed best among the derived markers in our series (AUC 0.786) is consistent with these studies; our lower AUC compared with the values reported at diagnosis may be explained by the first-trimester timing of our measurements. The cut-off of 1.06 reported by Eyisoy et al. and our value of 0.245 are not directly comparable: the former aims to discriminate severity among women with ICP in the third trimester, whereas the latter reflects the difference between women who later develop ICP and healthy controls in the first trimester. Moreover, the association between APRI at diagnosis and bile acids and perinatal outcomes reported by Eyisoy et al. was not observed in our series, a difference consistent with the interpretation that first-trimester APRI reflects early hepatocellular susceptibility rather than disease severity.

The absence of significant findings for FIB-4 in any analysis is not unexpected and stems from the structure of the formula. FIB-4 contains the square root of ALT in its denominator and age in its numerator [21]. Hepatocellular injury in ICP is ALT-predominant, as reflected in the significantly lower AST/ALT ratio in our ICP group (median 0.83 vs. 1.27; *p* < 0.001); the rise in ALT therefore mathematically dampens the contribution of the rise in AST to the index. In addition, the discriminative power of this score, developed in populations with chronic liver disease, depends largely on age, a component that remains nearly constant in a young pregnant population with a narrow age range such as ours. The absence of ALT from the APRI denominator, and its reliance on the direct AST/platelet relationship, explains why it discriminates much better in the same population.

Two points should be emphasised in interpreting the discrimination findings. First, first-trimester ALT alone discriminated ICP better than any derived index (AUC 0.895), and adding APRI and SII to ALT did not significantly improve discrimination; adjustment for ALT also attenuated the associations of APRI and SII to borderline significance. The finding that ALT exceeded 40 U/L in the first trimester in more than one third of women with ICP suggests that, in some of these women, ICP may be associated with a subclinical hepatocellular process beginning well before clinical manifestations, but also that coexisting conditions such as undiagnosed fatty liver disease may have contributed. The discriminative value of APRI thus derives largely from its AST component, with only a limited contribution from the haemogram component. Second, selecting controls with normal liver tests inflated the discrimination of transaminase-based markers (spectrum bias) [22,23]: when cases were restricted to the transaminase range of controls, the AUC of APRI fell from 0.786 to 0.644, to the level of SII and NLR. In a population including women with mildly elevated transaminases from other causes, the true discrimination of APRI would therefore be expected to be considerably lower than the main estimate. Furthermore, in a case–control design, PPV and NPV depend on the proportion of cases in the sample; the PPV of 85.6% falls to 3.2% at a population prevalence of 1%. The use of first-trimester APRI for screening is therefore not supported by the present data, and we have accordingly framed the study as one of association rather than of diagnostic accuracy.

The most important negative finding of the study is that first-trimester indices did not demonstrate an association with perinatal outcome. This is not surprising from a pathophysiological standpoint: fetal harm in ICP results from the direct effects of bile acids crossing the placenta on fetal cardiomyocytes and the myometrium rather than from the maternal inflammatory burden [2]. In the aggregate data meta-analysis of 5557 cases from 23 studies and the individual patient data meta-analysis of 5269 cases from 27 studies by Ovadia et al., the stillbirth rate in singleton pregnancies increased from 0.13% in women with bile acids <40 µmol/L to 3.44% in those with ≥100 µmol/L, with a significant increase in risk demonstrated only in the latter group [3]. The meta-analysis by Cui et al. reported a graded relationship between increasing bile acid concentrations and the risks of preterm birth, meconium-stained amniotic fluid, and NICU admission [37]. Çelik et al. found that preterm birth and NICU admission increased above a cut-off of approximately 34 µmol/L in 370 women with ICP [38], and Marschall’s review emphasised that fetal risk becomes evident above 40 µmol/L [39]. In our series, the most informative estimate of the bile acid–outcome association is the one for the composite excluding preterm birth (AUC 0.681; adjusted OR 1.43 per 10 µmol/L), because women with bile acids ≥40 µmol/L were delivered before 37 weeks according to institutional protocol; the 100% rates of preterm birth, and hence of the full composite outcome, in the moderate and severe groups are largely definitional, and the Youden-derived cut-off of ≥39 µmol/L with its specificity of 98.4% essentially reproduces the protocol threshold. No association was observed among women delivered at 37 weeks with bile acids <40 µmol/L. These findings indicate that the association between bile acids and perinatal risk is genuine, but that its magnitude in our series would be inflated by bile acid-based decisions on the timing of delivery if the full composite were used. The sensitivity of only 44–47% also suggests that a low bile acid concentration does not in itself provide reassurance. The graded deterioration across severity subgroups—the decline in median gestational age at delivery from 37.0 to 34.7 weeks and preterm birth rates of 100% in the moderate and severe groups—is consistent with guideline recommendations for severity-based surveillance and timing of delivery [4,20,40]. The persistence of the severity-related reduction in birth weight after adjustment for gestational age suggests a fetal effect beyond earlier delivery in severe disease, but with only six women in the severe group, this observation remains hypothesis-generating.

The inverse association of SII with preterm birth (AUC 0.354; adjusted OR 0.51 per SD) arose in an exploratory analysis, did not survive Bonferroni correction, and was only borderline after FDR correction (q = 0.045–0.050). It was not observed for the co-primary outcomes, and preterm birth in this series was largely protocol-driven. We therefore regard it as a probable chance finding and do not interpret it further.

From a therapeutic perspective, our findings support the current approach whereby surveillance remains centred on bile acids. The individual participant data meta-analysis by Ovadia et al. showed that ursodeoxycholic acid confers only limited benefit on perinatal outcomes, particularly when used alone and in mild disease [41], and the PITCHES trial by Chappell et al., which randomised 605 women, found no difference in the composite perinatal outcome between ursodeoxycholic acid and placebo [42]. The Cochrane review by Walker et al. likewise concluded that the evidence on the effect of current pharmacological interventions on perinatal outcomes is insufficient [43]. In the absence of an effective pharmacological option, risk stratification and timing of delivery remain the mainstay of clinical management; this makes the search for a marker that could replace bile acid measurement all the more relevant, but our data indicate that haemogram-based indices cannot fill this gap.

The limited predictive power of first-trimester indices is consistent with their performance in other obstetric applications. Cevher Akdulum et al. examined the association of first-trimester SII with pre-eclampsia in 130 pregnant women and obtained only moderate predictive performance [44]. Karataş et al., investigating the value of AISI, NLR, SII and SIRI calculated at 11–14 weeks for predicting gestational diabetes and related obstetric outcomes, similarly reported limited discrimination [45]. These two studies show that first-trimester haemogram indices are generally significant at the group level but insufficient at the individual level for pregnancy complications, and our results confirm this pattern for ICP. It should also be borne in mind that coexisting metabolic disease increases risk: in the multicentre cohort of 2178 women with ICP reported by Li et al., coexisting gestational diabetes significantly aggravated adverse perinatal outcomes [46].

The higher BMI in the control group and its independent inverse association with ICP in the multivariable model (OR 0.65 per SD; *p* = 0.027) should be regarded as a design-related finding rather than a biological protective effect. The selection of controls from women followed at the same centre and the median BMI above 28 kg/m^2^ in both groups point to a high weight profile across the whole population, in which a difference of 1.3–1.7 kg/m^2^ is of limited clinical relevance. Indeed, BMI was not an independent predictor in any model of perinatal outcome (all *p* > 0.15), and all effect sizes remained practically unchanged after adjustment for BMI and age, indicating that the main findings are robust to confounding.

## 5. Strengths and Limitations

The main strength of this study is that the indices were evaluated before disease onset, in the first trimester; most previous studies performed measurements at diagnosis, thereby blurring the distinction between prediction and diagnosis. Additional methodological strengths include the evaluation of both the group-level discrimination and the perinatal outcome question in the same cohort, the three-category bile acid-based severity subgroup analysis, adjustment for BMI and age in all models, bootstrap estimation of AUC confidence intervals, explicit control of multiplicity, a quantitative assessment of spectrum bias, and sensitivity analyses addressing the diagnostic threshold and the unequal group sizes.

Several limitations should be acknowledged. First, the study was single-centre and retrospective, so incomplete record-based information and selection bias cannot be fully excluded. Second, the case–control design with controls selected for normal liver tests introduced spectrum bias, which we quantified but could not remove; the QUADAS-2 appraisal rated the patient-selection domain at high risk of bias (Supplementary Table S3). Asymptomatic controls did not undergo bile acid measurement, so subclinical hypercholanaemia cannot be excluded among them (differential verification). Owing to the case–control design and the unequal group sizes (115 vs. 64), PPV and NPV cannot be generalised to the population level, and the cut-offs reported here are data-derived and require external validation. Third, the within-ICP analyses were adequately powered for effects of AUC 0.65 but had limited power for smaller effects; the finding that the indices did not demonstrate an association with perinatal outcome therefore does not exclude a small association. The limitation of the severe subgroup to six women also means that the proportions in this group carry wide uncertainty. Fourth, the composite outcome weights components of differing clinical importance (such as NICU admission and fetal distress) equally. Fifth, and perhaps most importantly, because delivery was planned at 37 weeks in women with ICP, and earlier when bile acids were ≥40 µmol/L, according to institutional protocol, and because the mode of labour onset was not recorded, preterm births could not be classified as spontaneous or iatrogenic; part of the strong association between bile acids and preterm birth therefore stems from the clinical decision-making process, which is why the composite excluding preterm birth was treated as co-primary. Sixth, a threshold of ≥10 µmol/L was used for the diagnosis of ICP; when the ≥19 µmol/L threshold of the RCOG (28) was applied in a sensitivity analysis, 26 women (22.6%) were reclassified, but the results were materially unchanged. Seventh, although first-trimester ALT exceeded 40 U/L in 35.7% of women with ICP, imaging data for hepatic steatosis and comprehensive hepatitis serology could not be included in the analyses; a contribution of coexisting subclinical liver disease therefore cannot be excluded. Eighth, the dose, timing of initiation and response to ursodeoxycholic acid, as well as neonatal sex, were not available in the records and could not be analysed, and birth weight could not be expressed as a percentile. Finally, no stillbirths occurred in our series; our findings therefore cannot be generalised to the most severe outcome of ICP. Multicentre prospective cohorts with serial measurement of the indices throughout pregnancy, unselected controls and detailed recording of delivery timing are needed to address these questions.

## 6. Conclusions

First-trimester APRI, calculated from routine haemogram and liver tests at 11–14 weeks of gestation, is higher months before clinical presentation in women who subsequently develop ICP and shows the strongest group-level association among the derived markers (AUC 0.786); SII and NLR show statistically significant but small associations, and FIB-4 and PLR show none. However, these indices add nothing to the discrimination provided by first-trimester ALT, and the apparent accuracy of APRI is substantially inflated by the selection of controls with normal liver tests. These findings support the view that, in a subset of women, ICP may be associated with a hepatocellular–inflammatory process beginning in early pregnancy, but they do not support the use of these indices for screening.

In contrast, none of the first-trimester indices demonstrated an association with adverse perinatal outcome, preterm birth or NICU admission, or was related to disease severity or correlated with serum bile acids, although small associations cannot be excluded with the present sample size. Serum bile acid concentration is the only independent variable associated with perinatal risk, although the magnitude of this association is influenced by the bile acid-based delivery-timing protocol. First-trimester APRI and SII should therefore remain research-stage early risk markers until validated in prospective studies, and decisions on the intensity of surveillance, frequency of antenatal visits and timing of delivery in ICP should continue to be based on bile acid measurement.

## Figures and Tables

**Table 1:** Demographic, obstetric and first-trimester laboratory characteristics of the ICP and control groups.

Variable	ICP (*n* = 115)	Control (*n* = 64)	*p*	q
**A. Demographic and obstetric characteristics**				
Age (years)	30.0 (26.0–33.0)	29.0 (25.0–34.0)	0.469	0.503
Gravidity	2 (1–3)	2 (1–3)	0.827	0.827
Parity	1 (0–1)	1 (0–1)	0.172	0.215
Body mass index (kg/m^2^)	28.4 (27.0–30.5)	30.1 (28.6–32.0)	0.004	0.009
**B. Laboratory parameters (11–14 weeks)**				
AST (U/L)	25.0 (19.5–33.0)	17.0 (13.0–21.2)	<0.001	<0.001
ALT (U/L)	33.0 (20.5–45.5)	13.0 (12.0–15.0)	<0.001	<0.001
AST/ALT ratio	0.83 (0.62–0.97)	1.27 (0.93–1.64)	<0.001	<0.001
Platelets (×10^9^/L)	230.0 (196.0–254.0)	226.0 (194.5–311.2)	0.170	0.215
Neutrophils (×10^9^/L)	6.37 (5.63–7.48)	6.00 (5.80–6.20)	0.026	0.049
Lymphocytes (×10^9^/L)	1.98 (1.67–2.50)	1.98 (1.94–2.70)	0.130	0.194
**C. Derived inflammatory and fibrosis indices**				
NLR	3.20 (2.80–3.66)	2.96 (2.24–3.18)	0.002	0.005
PLR	115.2 (94.9–131.0)	115.0 (106.1–126.9)	0.455	0.503
SII (×10^9^/L) ^†^	747.8 (638.0–851.2)	686.7 (655.5–750.2)	0.011	0.024
APRI ^†^	0.285 (0.218–0.376)	0.178 (0.127–0.238)	<0.001	<0.001
FIB-4	0.60 (0.47–0.72)	0.53 (0.40–0.70)	0.104	0.174

Values are median (interquartile range). Because no continuous variable was normally distributed in both groups (Shapiro–Wilk test), all comparisons were performed with the Mann–Whitney U test. q: Benjamini–Hochberg false discovery rate-adjusted *p*-value across the 15 comparisons in this table. ^†^ Primary hypotheses, evaluated at a Bonferroni-corrected α of 0.025. ICP: intrahepatic cholestasis of pregnancy; NLR: neutrophil-to-lymphocyte ratio; PLR: platelet-to-lymphocyte ratio; SII: systemic immune-inflammation index (neutrophils × platelets/lymphocytes); APRI: AST-to-platelet ratio index; FIB-4: fibrosis-4 index.

**Table 2:** Perinatal outcomes by group.

Variable	ICP (*n* = 115)	Control (*n* = 64)	Crude OR (95% CI)	*p*	Adjusted OR (95% CI) ^a^	*p*
**A. Continuous variables, median (IQR)**						
Gestational age at delivery (weeks)	37.0 (36.3–37.0)	38.0 (37.0–39.0)	—	<0.001 ^b^	—	—
Birth weight (g)	2950 (2750–3120)	3175 (2973–3413)	—	<0.001 ^b^	—	—
1-min Apgar score	8 (7–8)	8 (8–8)	—	0.002 ^b^	—	—
**B. Categorical outcomes, *n* (%)**						
Preterm birth (<37 weeks)	39 (33.9)	10 (15.6)	2.77 (1.27–6.03)	0.010	2.71 (1.23–6.00)	0.014
Meconium-stained amniotic fluid	16 (13.9)	4 (6.3)	2.42 (0.77–7.59)	0.128	2.26 (0.70–7.22)	0.171
Fetal distress	16 (13.9)	7 (10.9)	1.32 (0.51–3.39)	0.569	1.15 (0.43–3.04)	0.781
NICU admission	27 (23.5)	15 (23.4)	1.00 (0.49–2.06)	0.995	0.93 (0.44–1.96)	0.856
1-min Apgar score < 7	5 (4.3)	3 (4.7)	0.92 (0.21–4.00)	0.916	0.95 (0.21–4.30)	0.947
Composite adverse perinatal outcome	52 (45.2)	21 (32.8)	1.69 (0.89–3.20)	0.107	1.56 (0.81–3.01)	0.182
Composite excluding preterm birth	32 (27.8)	16 (25.0)	1.16 (0.58–2.32)	0.683	1.09 (0.53–2.24)	0.812

Dependent variable in each logistic model: the respective outcome (present = 1, absent = 0); independent variable: group (ICP = 1; control = 0, reference). Crude and adjusted ORs, their 95% CIs, and *p*-values (Wald) are all obtained from the same logistic regression models. ^a^ Adjusted for age and BMI. ^b^ Mann–Whitney U test. The composite adverse perinatal outcome was defined as the presence of at least one of the following: preterm birth (<37 weeks), meconium-stained amniotic fluid, fetal distress, NICU admission, or 1-min Apgar score < 7. OR: odds ratio; CI: confidence interval; IQR: interquartile range; NICU: neonatal intensive care unit.

**Table 3:** ROC analyses: discrimination of markers for ICP and association with adverse perinatal outcomes.

Marker	AUC (95% CI)	*p*	q	Cut-Off	Sensitivity (%)	Specificity (%)	PPV (%)	NPV (%)
**A. ICP vs. controls (*n* = 179; 115 vs. 64)**								
ALT	0.895 (0.843–0.940)	<0.001	<0.001	≥20.0	75.7	96.9	97.8	68.9
APRI ^†^	0.786 (0.718–0.852)	<0.001	primary	≥0.245	67.0	79.7	85.6	57.3
AST	0.782 (0.714–0.846)	<0.001	<0.001	≥21.0	72.2	68.8	80.6	57.9
AST/ALT ratio (inverse)	0.787 (0.712–0.853)	<0.001	<0.001	≤0.977	75.7	73.4	83.7	62.7
NLR	0.642 (0.552–0.726)	0.002	0.011	≥3.189	55.7	75.0	80.0	48.5
SII ^†^	0.615 (0.530–0.697)	0.011	primary	≥713.8	65.2	71.9	80.6	53.5
FIB-4	0.573 (0.484–0.662)	0.104	0.269	—	—	—	—	—
PLR	0.466 (0.382–0.548)	0.455	0.740	—	—	—	—	—
**B. Co-primary: composite adverse perinatal outcome in women with ICP (*n* = 115; 52 events)**								
Bile acids	0.754 (0.656–0.842)	<0.001	<0.001	≥39.0 ^‡^	44.2	98.4	95.8	68.1
SII	0.412 (0.307–0.520)	0.105	0.269	—	—	—	—	—
NLR	0.464 (0.361–0.572)	0.504	0.757	—	—	—	—	—
PLR	0.543 (0.437–0.651)	0.428	0.726	—	—	—	—	—
APRI	0.466 (0.359–0.571)	0.527	0.762	—	—	—	—	—
FIB-4	0.565 (0.457–0.669)	0.230	0.465	—	—	—	—	—
**C. Co-primary: composite excluding preterm birth in women with ICP (*n* = 115; 32 events)**								
Bile acids	0.681 (0.556–0.796)	0.003	0.015	≥41.0	46.9	91.6	68.2	81.7
SII	0.517 (0.400–0.634)	0.776	0.835	—	—	—	—	—
NLR	0.470 (0.362–0.585)	0.622	0.835	—	—	—	—	—
PLR	0.523 (0.409–0.636)	0.701	0.835	—	—	—	—	—
APRI	0.405 (0.294–0.527)	0.117	0.269	—	—	—	—	—
FIB-4	0.482 (0.362–0.601)	0.762	0.835	—	—	—	—	—
**D. Exploratory: preterm birth (<37 weeks) in women with ICP (*n* = 115; 39 events)**								
Bile acids	0.843 (0.756–0.917)	<0.001	<0.001	≥39.0 ^‡^	59.0	98.7	95.8	82.4
SII	0.354 (0.254–0.456)	0.010	0.045	—	—	—	—	—
NLR	0.462 (0.352–0.572)	0.504	0.757	—	—	—	—	—
PLR	0.522 (0.417–0.628)	0.697	0.835	—	—	—	—	—
APRI	0.552 (0.434–0.662)	0.361	0.641	—	—	—	—	—
FIB-4	0.578 (0.462–0.691)	0.173	0.375	—	—	—	—	—
**E. Exploratory: NICU admission in women with ICP (*n* = 115; 27 events)**								
Bile acids	0.678 (0.540–0.806)	0.005	0.025	≥41.0	51.9	90.9	63.6	86.0
SII	0.481 (0.364–0.603)	0.769	0.835	—	—	—	—	—
NLR	0.483 (0.371–0.596)	0.792	0.835	—	—	—	—	—
PLR	0.471 (0.351–0.585)	0.647	0.835	—	—	—	—	—
APRI	0.429 (0.314–0.554)	0.269	0.500	—	—	—	—	—
FIB-4	0.520 (0.404–0.638)	0.759	0.835	—	—	—	—	—

All AUCs are given in the a priori hypothesised direction (higher marker values indicating a higher probability of the event; lower values for the AST/ALT ratio); AUC < 0.5 indicates an inverse association, and no curve was re-oriented post hoc. *p*-values test AUC = 0.5 (Mann–Whitney U test). q: Benjamini–Hochberg FDR-adjusted *p* across the 39 secondary tests listed in Supplementary Table S4. ^†^ Primary hypotheses, α = 0.025. Cut-offs (Youden index) are reported only where the AUC differed significantly from 0.5 in the hypothesised direction. ^‡^ The bile acid cut-off reproduces the institutional ≥40 µmol/L delivery-timing threshold, so its specificity for outcomes that include preterm birth is largely definitional. 95% CIs were computed with 4000 bootstrap replicates. AUC comparisons (DeLong test): ALT vs. APRI *p* = 0.003; APRI vs. AST/ALT ratio *p* = 0.992; APRI vs. SII *p* = 0.004; APRI vs. NLR *p* = 0.001. PPV and NPV were calculated for the proportion of ICP in the sample (64.2%); at a population prevalence of 1%, the PPV and NPV of APRI ≥0.245 are 3.2% and 99.6%, respectively. AUC: area under the curve; PPV/NPV: positive/negative predictive value; NICU: neonatal intensive care unit.

**Table 4:** Comparison of women with and without the composite adverse perinatal outcome within the ICP group.

Variable	Adverse Outcome (*n* = 52)	No Adverse Outcome (*n* = 63)	*p*
Age (years)	31.0 (26.0–35.0)	28.0 (26.0–33.0)	0.119
BMI (kg/m^2^)	28.4 (27.0–30.1)	28.4 (27.0–30.8)	0.917
Bile acids (µmol/L)	34.0 (23.0–49.5)	21.0 (17.0–28.0)	<0.001
AST (U/L)	23.5 (18.0–33.0)	26.0 (21.0–33.5)	0.321
ALT (U/L)	26.0 (18.0–43.0)	35.0 (22.5–46.0)	0.104
Platelets (×10^9^/L)	219.5 (197.8–247.8)	235.0 (194.0–260.5)	0.455
NLR	3.21 (2.81–3.55)	3.20 (2.80–3.75)	0.504
PLR	119.3 (97.9–129.0)	105.9 (93.0–132.4)	0.428
SII (×10^9^/L)	739.8 (620.6–824.9)	761.4 (684.4–865.8)	0.105
APRI	0.278 (0.224–0.364)	0.301 (0.218–0.386)	0.527
FIB-4	0.60 (0.50–0.78)	0.60 (0.46–0.68)	0.230
Gestational age at delivery (weeks)	36.0 (36.0–36.9)	37.0 (37.0–37.0)	<0.001
Birth weight (g)	2805 (2600–3000)	3000 (2900–3200)	<0.001

Values are median (interquartile range); all comparisons were made by the Mann–Whitney U test. BMI: body mass index; NLR: neutrophil-to-lymphocyte ratio; PLR: platelet-to-lymphocyte ratio; SII: systemic immune-inflammation index; APRI: AST-to-platelet ratio index; FIB-4: fibrosis-4 index.

**Table 5:** Disease severity groups according to serum bile acids.

Variable	Mild (<40 µmol/L) *n* = 93	Moderate (40–99 µmol/L) *n* = 16	Severe (≥100 µmol/L) *n* = 6	*p*
**Continuous variables: median (IQR) for mild and moderate; median (range) for severe**				
Bile acids (µmol/L)	23.0 (18.0–29.0)	48.0 (42.8–56.0)	135.5 (111–142)	<0.001
AST (U/L)	24.0 (19.0–33.0)	31.0 (23.8–36.5)	32.0 (10–59)	0.072
ALT (U/L)	33.0 (19.0–45.0)	39.0 (25.0–49.2)	71.0 (24–128)	0.024
Platelets (×10^9^/L)	231 (199–255)	212.5 (190.8–271.0)	232.5 (187–245)	0.872
NLR	3.20 (2.78–3.73)	3.29 (3.04–3.56)	3.15 (2.82–3.71)	0.816
SII (×10^9^/L)	751.8 (624.4–859.4)	754.8 (664.6–842.7)	693.0 (618.9–741.1)	0.349
APRI	0.272 (0.212–0.363)	0.343 (0.298–0.378)	0.364 (0.102–0.628)	0.083
FIB-4	0.60 (0.47–0.71)	0.76 (0.58–0.89)	0.54 (0.15–0.66)	0.054
Gestational age at delivery (weeks)	37.0 (37.0–37.0)	36.0 (36.0–36.0)	34.7 (32.0–36.0)	<0.001
Birth weight (g)	3000 (2850–3200)	2750 (2526–2850)	2350 (1450–2440)	<0.001
1-min Apgar score	8 (7–8)	7 (7–8)	7 (6–8)	0.002
**Perinatal outcomes, *n* (%) (Wilson 95% CI)**				
Preterm birth	17 (18.3) 11.7–27.3	16 (100.0) 80.6–100.0	6 (100.0) 61.0–100.0	<0.001
Meconium-stained amniotic fluid	7 (7.5) 3.7–14.7	5 (31.3) 14.2–55.6	4 (66.7) 30.0–90.3	<0.001
Fetal distress	5 (5.4) 2.3–12.0	7 (43.8) 23.1–66.8	4 (66.7) 30.0–90.3	<0.001
NICU admission	13 (14.0) 8.4–22.5	10 (62.5) 38.6–81.5	4 (66.7) 30.0–90.3	<0.001
1-min Apgar score < 7	0 (0.0) 0.0–4.0	3 (18.8) 6.6–43.0	2 (33.3) 9.7–70.0	<0.001
Composite adverse outcome	30 (32.3) 23.6–42.3	16 (100.0) 80.6–100.0	6 (100.0) 61.0–100.0	<0.001
Composite excluding preterm birth	17 (18.3) 11.7–27.3	11 (68.8) 44.4–85.8	4 (66.7) 30.0–90.3	<0.001

Continuous variables: Kruskal–Wallis test with Bonferroni correction for pairwise comparisons; perinatal outcomes: chi-square/Fisher–Freeman–Halton test. Because the severe group comprises only six women, its estimates are imprecise and hypothesis-generating; individual values are provided in Supplementary Table S1. IQR: interquartile range; NICU: neonatal intensive care unit.

**Table 6:** Logistic regression analyses.

Variable	Univariable OR (95% CI)	*p*	Multivariable OR (95% CI)	*p*
**A. Dependent variable: ICP (=1) vs. control (=0, reference); *n* = 179; model *p* < 0.001; Nagelkerke R^2^ = 0.380; Hosmer–Lemeshow χ^2^ = 7.97, *p* = 0.436**				
Age (per SD, 5.3 years)	1.19 (0.87–1.63)	0.265	1.38 (0.95–2.00)	0.089
BMI (per SD, 3.3 kg/m^2^)	0.66 (0.48–0.91)	0.011	0.65 (0.44–0.95)	0.027
APRI (per SD, 0.129)	4.49 (2.62–7.70)	<0.001	4.79 (2.70–8.50)	<0.001
SII (per SD, 131.6 × 10^9^/L)	1.32 (0.97–1.81)	0.079	1.64 (1.11–2.43)	0.014
NLR (per SD, 0.84)	1.53 (1.09–2.15)	0.014	—	—
**B. Co-primary—dependent variable: composite adverse perinatal outcome (present = 1 vs. absent = 0, reference), ICP group; *n* = 115; model *p* < 0.001; Nagelkerke R^2^ = 0.384; Hosmer–Lemeshow χ^2^ = 8.36, *p* = 0.399**				
Bile acids (per 10 µmol/L)	2.29 (1.52–3.47)	<0.001	2.38 (1.54–3.69)	<0.001
APRI (per SD, 0.133)	0.95 (0.65–1.37)	0.767	0.75 (0.47–1.21)	0.239
SII (per SD, 149.0 × 10^9^/L)	0.73 (0.50–1.07)	0.103	0.68 (0.44–1.06)	0.092
FIB-4 (per SD, 0.244)	1.40 (0.94–2.08)	0.097	—	—
NLR (per SD, 0.81)	0.77 (0.53–1.13)	0.187	—	—
Age (per SD, 5.3 years)	1.39 (0.95–2.03)	0.092	1.42 (0.91–2.20)	0.122
BMI (per SD, 3.1 kg/m^2^)	1.01 (0.70–1.47)	0.941	1.00 (0.64–1.56)	0.981
**C. Co-primary—dependent variable: composite excluding preterm birth (present = 1 vs. absent = 0, reference), ICP group; *n* = 115; model *p* = 0.001; Nagelkerke R^2^ = 0.234; Hosmer–Lemeshow χ^2^ = 5.88, *p* = 0.660**				
Bile acids (per 10 µmol/L)	1.34 (1.11–1.62)	0.002	1.43 (1.16–1.76)	0.001
APRI (per SD, 0.133)	0.70 (0.44–1.12)	0.139	0.56 (0.315–0.995)	0.048^§^
SII (per SD, 149.0 × 10^9^/L)	1.00 (0.67–1.51)	0.986	1.01 (0.64–1.59)	0.978
Age (per SD, 5.3 years)	1.28 (0.85–1.92)	0.237	1.37 (0.87–2.15)	0.178
BMI (per SD, 3.1 kg/m^2^)	1.09 (0.72–1.63)	0.695	1.04 (0.65–1.66)	0.870
**D. Exploratory—dependent variable: preterm birth (present = 1 vs. absent = 0, reference), ICP group; *n* = 115; model *p* < 0.001; Nagelkerke R^2^ = 0.551; Hosmer–Lemeshow χ^2^ = 4.70, *p* = 0.789**				
Bile acids (per 10 µmol/L)	3.53 (2.08–5.99)	<0.001	3.89 (2.18–6.91)	<0.001
SII (per SD, 149.0 × 10^9^/L)	0.65 (0.43–0.96)	0.032	0.51 (0.30–0.87)	0.014 ^§^
APRI (per SD, 0.133)	1.28 (0.87–1.88)	0.210	1.01 (0.59–1.73)	0.982
BMI (per SD, 3.1 kg/m^2^)	1.03 (0.70–1.52)	0.872	1.10 (0.65–1.86)	0.728
Age (per SD, 5.3 years)	1.10 (0.75–1.62)	0.617	1.03 (0.62–1.71)	0.912

All predictors are continuous; ORs express the change in the odds of the dependent variable (coded 1) relative to its reference category (coded 0) per 1-SD increase (SD shown in parentheses; whole sample for Panel A, ICP group for Panels B–D) or, for bile acids, per 10 µmol/L increase. Univariable ORs are from separate models containing one predictor each; multivariable ORs are from the single model shown in each panel (Panel A: age, BMI, APRI and SII; Panels B–D: bile acids, APRI, SII, age and BMI). NLR was not entered into the main models because it is a mathematical component of SII; variables analysed univariably but not entered into the model are shown as “—”. All VIF ≤ 1.08. ^§^ Not significant after FDR correction (q = 0.156 for APRI in Panel C and 0.050 for SII in Panel D; Supplementary Table S4). OR: odds ratio; CI: confidence interval; SD: standard deviation; BMI: body mass index; VIF: variance inflation factor.

## Data Availability

The anonymised dataset supporting this study is available from the corresponding author upon reasonable request; the analysis code is available from the authors on request.

## Supplementary Materials

The following supporting information can be downloaded at: https://www.mdpi.com/article/10.3390/diagnostics16213610/s1. Table S1: Individual values of the six women with severe ICP; Table S2: Sensitivity analyses; Table S3: QUADAS-2 risk-of-bias appraisal; Table S4: Secondary inferential tests with Benjamini–Hochberg q values.
