# Supplementary material for: First-Trimester Systemic Immune-Inflammation Index, APRI and FIB-4 Scores in Women Who Later Develop Intrahepatic Cholestasis of Pregnancy: Association with the Disease and with Perinatal Outcomes in a Retrospective Case–Control Study
Source: Diagnostics (Basel). 2026 Nov 1;16(21):3610. doi: 10.3390/diagnostics16213610 (PMC13761477; doi:10.3390/diagnostics16213610)
Supplement: Supplementary file 1 [file diagnostics-16-03610-s001.zip › diagnostics-4627663-supplementary.pdf]

Supplementary Materials

First-Trimester Systemic Immune-Inflammation Index, APRI and FIB-4 Scores in Women Who Later Develop Intrahepatic Cholestasis of Pregnancy: Association with the Disease and with Perinatal Outcomes in a Retrospective Case–Control Study

Mukremin Ceylan, Mucahit Furkan Balcı, Abdulmecit Öktem, Raziye Torun, Orhan Nural and Hakan Golbasi

Table S1. Individual first-trimester laboratory values and perinatal outcomes of the six women with severe ICP (bile acids ≥100 µmol/L), ordered by bile acid concentration.

Bile acids (µmol/L)	ALT (U/L)	AST (U/L)	PLT (×10⁹/L)	NLR	SII (×10⁹/L)	APRI	FIB-4	GA at delivery (wk+d)	Birth weight (g)	1-min Apgar	Adverse outcomes
111	85	42	187	3.71	692.9	0.561	0.658	35+5	2300	8	Preterm birth
121	85	42	230	3.22	741.1	0.457	0.535	35+3	2300	8	Preterm birth
134	36	22	235	2.82	661.9	0.234	0.406	34+0	2440	6	Preterm birth, MSAF, fetal distress, NICU, Apgar <7
137	24	21	194	3.19	618.9	0.271	0.552	32+0	1450	6	Preterm birth, MSAF, fetal distress, NICU, Apgar <7
141	57	10	245	2.83	693.1	0.102	0.146	36+0	2400	7	Preterm birth, MSAF, fetal distress, NICU
142	128	59	235	3.12	732.4	0.628	0.621	34+0	2440	7	Preterm birth, MSAF, fetal distress, NICU

Laboratory values are first-trimester (11–14 weeks) measurements; bile acids were measured at diagnosis. All six deliveries were preterm in accordance with the institutional protocol for bile acids ≥40 µmol/L. ALT: alanine aminotransferase; AST: aspartate aminotransferase; PLT: platelet count; NLR: neutrophil-to-lymphocyte ratio; SII: systemic immune-inflammation index; APRI: AST-to-platelet ratio index; FIB-4: fibrosis-4 index; GA: gestational age; MSAF: meconium-stained amniotic fluid; NICU: neonatal intensive care unit admission.

Table S2. Sensitivity analyses.

Analysis	Cases/controls	APRI	SII	NLR	ALT
**A. Discrimination of ICP from controls: AUC (95% CI)**					
Main analysis	115/64	0.786 (0.718–0.852)	0.615 (0.530–0.697)	0.642 (0.552–0.726)	0.895 (0.843–0.940)
(i) ICP redefined as bile acids ≥19 µmol/L (RCOG)	89/64	0.782 (0.708–0.849)	0.597 (0.508–0.689)	0.648 (0.555–0.738)	0.897 (0.839–0.945)
(ii) Transaminase-restricted: cases with ALT ≤31 U/L (range of controls)	50/64	0.644 (0.541–0.741)	0.621 (0.508–0.738)	0.641 (0.534–0.742)	0.759 (0.656–0.851)
(ii-b) Cases with ALT ≤40 U/L (upper limit of normal)	74/64	0.705 (0.615–0.790)	0.569 (0.468–0.670)	0.609 (0.516–0.704)	0.837 (0.761–0.905)
(iv) 2000 random 1:1 subsamples, median [2.5th–97.5th percentile]	64/64	0.787 [0.746–0.826]	0.614 [0.557–0.675]	0.642 [0.599–0.684]	0.896 [0.857–0.936]
Proportion of subsamples with $p < 0.05$		100%	66.9%	97.3%	100%
**B. Multivariable models of ICP presence: OR per 1-SD increase (95% CI); *p***					

Analysis	Cases/controls	APRI	SII	NLR	ALT
Main model (age, BMI, APRI, SII)	115/64	4.79 (2.70–8.50); $p < 0.001$	1.64 (1.11–2.43); $p = 0.014$	—	—
(iii) Main model + ALT (Hosmer–Lemeshow $p = 0.159$; VIF ≤ 2.36)	115/64	2.21 (1.00–4.87); $p = 0.049$	1.68 (1.00–2.83); $p = 0.050$	—	209 (26–1704); $p < 0.001$
(i) RCOG ≥19 µmol/L definition	89/64	4.60 (2.52–8.42); $p < 0.001$	1.59 (1.04–2.44); $p = 0.031$	—	—
(ii) Transaminase-restricted, + ALT	50/64	2.17 (0.99–4.80); $p = 0.054$	1.74 (1.02–2.95); $p = 0.041$	—	112 (11–1170); $p < 0.001$
(iv) 1:1 subsamples: median OR; % with $p < 0.05$	64/64	4.95; 100%	1.73; 68.2%	—	—

**Outcome within ICP (RCOG ≥19 µmol/L definition; *n* = 89)**	**Events**	**Bile acids AUC (95% CI); *p***	**SII AUC (95% CI); *p***	**APRI AUC (95% CI); *p***	**FIB-4 AUC (95% CI); *p***
Composite adverse perinatal outcome	46	0.761 (0.658–0.853); $p < 0.001$	0.405 (0.292–0.529); $p = 0.126$	0.532 (0.414–0.653); $p = 0.605$	0.609 (0.491–0.725); $p = 0.077$
Composite excluding preterm birth	28	0.684 (0.546–0.811); $p = 0.006$	0.527 (0.397–0.652); $p = 0.688$	0.448 (0.323–0.581); $p = 0.437$	0.513 (0.376–0.649); $p = 0.842$

AUCs are given in the a priori hypothesised direction (AUC < 0.5 indicates an inverse association); 95% CIs were computed with 4000 bootstrap replicates. ORs are expressed per 1-SD increase of the whole sample. The very large ORs for ALT reflect near-complete separation, because all controls had ALT ≤31 U/L. RCOG: Royal College of Obstetricians and Gynaecologists; VIF: variance inflation factor; OR: odds ratio; CI: confidence interval.

Table S3. QUADAS-2 risk-of-bias and applicability appraisal of the discrimination analysis (ICP vs. controls).

QUADAS-2 domain	Risk of bias	Applicability concern	Rationale
Patient selection	High	High	Case–control (two-gate) design: consecutive cases, but controls randomly sampled from women with normal liver tests throughout pregnancy, which excludes women with mildly raised transaminases from other causes (spectrum bias). Several conditions were excluded in both groups.
Index test	High	Low	Indices were calculated from routine first-trimester values recorded before the diagnosis (blinded by timing), but cut-offs were derived from the same data (Youden index) and not prespecified, which inflates sensitivity and specificity.
Reference standard	Low	Low	ICP was defined by unexplained pruritus and fasting bile acids ≥10 μmol/L (SMFM/SOGC); results were robust to the RCOG ≥19 μmol/L threshold. The reference standard is independent of the index test.
Flow and timing	High	—	Asymptomatic controls did not undergo bile acid measurement (differential verification). Twenty-seven of 142 cases (19.0%) were excluded for missing data. The interval between index test (11–14 weeks) and diagnosis is inherent to the early-marker question.

QUADAS-2 is designed for diagnostic accuracy studies; it was applied here to make the limitations of the case–control design explicit. SMFM: Society for Maternal-Fetal Medicine; SOGC: Society of Obstetricians and Gynaecologists of Canada; RCOG: Royal College of Obstetricians and Gynaecologists.

Table S4. Secondary inferential tests (n = 39) with Benjamini–Hochberg false discovery rate (q) and Bonferroni-adjusted p values.

Test	*p*	*q* (BH)	*p* (Bonferroni)
ICP vs. controls — ROC, ALT	<0.001	<0.001	<0.001
ICP vs. controls — ROC, AST	<0.001	<0.001	<0.001
ICP vs. controls — ROC, AST/ALT ratio (inverse)	<0.001	<0.001	<0.001
ICP vs. controls — ROC, NLR	0.002	0.011	0.063
ICP vs. controls — ROC, FIB-4	0.104	0.269	1.000
ICP vs. controls — ROC, PLR	0.455	0.740	1.000
Composite adverse perinatal outcome — ROC, Bile acids	<0.001	<0.001	<0.001
Composite adverse perinatal outcome — ROC, SII	0.105	0.269	1.000
Composite adverse perinatal outcome — ROC, NLR	0.504	0.757	1.000
Composite adverse perinatal outcome — ROC, PLR	0.428	0.726	1.000
Composite adverse perinatal outcome — ROC, APRI	0.527	0.762	1.000
Composite adverse perinatal outcome — ROC, FIB-4	0.230	0.465	1.000
Composite excluding preterm birth — ROC, Bile acids	0.003	0.015	0.105
Composite excluding preterm birth — ROC, SII	0.776	0.835	1.000
Composite excluding preterm birth — ROC, NLR	0.622	0.835	1.000
Composite excluding preterm birth — ROC, PLR	0.701	0.835	1.000
Composite excluding preterm birth — ROC, APRI	0.117	0.269	1.000
Composite excluding preterm birth — ROC, FIB-4	0.762	0.835	1.000
Preterm birth — ROC, Bile acids	<0.001	<0.001	<0.001
Preterm birth — ROC, SII	0.010	0.045	0.407
Preterm birth — ROC, NLR	0.504	0.757	1.000
Preterm birth — ROC, PLR	0.697	0.835	1.000
Preterm birth — ROC, APRI	0.361	0.641	1.000
Preterm birth — ROC, FIB-4	0.173	0.375	1.000
NICU admission — ROC, Bile acids	0.005	0.025	0.203
NICU admission — ROC, SII	0.769	0.835	1.000
NICU admission — ROC, NLR	0.792	0.835	1.000

Test	*p*	*q* (BH)	*p* (Bonferroni)
NICU admission — ROC, PLR	0.647	0.835	1.000
NICU admission — ROC, APRI	0.269	0.500	1.000
NICU admission — ROC, FIB-4	0.759	0.835	1.000
ICP vs. controls — multivariable model, SII	0.014	0.050	0.545
Composite adverse perinatal outcome — multivariable model, APRI	0.239	0.465	1.000
Composite adverse perinatal outcome — multivariable model, SII	0.092	0.269	1.000
Composite excluding preterm birth — multivariable model, APRI	0.048	0.156	1.000
Composite excluding preterm birth — multivariable model, SII	0.978	0.982	1.000
Preterm birth — multivariable model, APRI	0.982	0.982	1.000
Preterm birth — multivariable model, SII	0.014	0.050	0.546
NICU admission — multivariable model, APRI	0.113	0.269	1.000
NICU admission — multivariable model, SII	0.649	0.835	1.000

The two primary hypotheses (APRI and SII discrimination of ICP vs. controls) were tested separately at $\alpha = 0.025$ (APRI $p < 0.001$; SII $p = 0.011$) and are not included in this family. ROC *p* values are from the Mann–Whitney U test; regression *p* values are Wald tests from the multivariable models (ICP vs. controls: age, BMI, APRI and SII; perinatal outcomes: bile acids, APRI, SII, age and BMI). BH: Benjamini–Hochberg; NICU: neonatal intensive care unit.
